# Machine Learning Prediction Models for Mechanically Ventilated Patients: Analyses of the MIMIC-III Database

**DOI:** 10.3389/fmed.2021.662340

**Published:** 2021-07-01

**Authors:** Yibing Zhu, Jin Zhang, Guowei Wang, Renqi Yao, Chao Ren, Ge Chen, Xin Jin, Junyang Guo, Shi Liu, Hua Zheng, Yan Chen, Qianqian Guo, Lin Li, Bin Du, Xiuming Xi, Wei Li, Huibin Huang, Yang Li, Qian Yu

**Affiliations:** ^1^Medical Research and Biometrics Center, National Center for Cardiovascular Diseases, Fuwai Hospital, Chinese Academy of Medical Sciences and Peking Union Medical College, Beijing, China; ^2^Department of Emergency, Guang'anmen Hospital, China Academy of Chinese Medical Sciences, Beijing, China; ^3^School of Economics and Management, Beijing Institute of Technology, Beijing, China; ^4^School of Computer Science and Technology, Wuhan University of Technology, Wuhan, China; ^5^Department of Burn Surgery, The First Affiliated Hospital of Naval Medical University, Shanghai, China; ^6^Translational Medicine Research Center, Fourth Medical Center and Medical Innovation Research Division of the Chinese People's Liberation Army (PLA) General Hospital, Beijing, China; ^7^Yidu Cloud Technology Inc., Beijing, China; ^8^Beijing Big Eye Xing Tu Culture Media Co., Ltd., Beijing, China; ^9^School of Information Science and Engineering, Hebei North University, Shijiazhuang, China; ^10^Medical ICU, Peking Union Medical College Hospital, Peking Union Medical College and Chinese Academy of Medical Sciences, Beijing, China; ^11^Department of Anesthesiology, Peking University Shougang Hospital, Beijing, China; ^12^Department of Critical Care Medicine, Fuxing Hospital, Capital Medical University, Beijing, China; ^13^Department of Critical Care Medicine, Beijing Tsinghua Changgung Hospital, School of Clinical Medicine, Tsinghua University, Beijing, China; ^14^Academy for Advanced Interdisciplinary Studies, Peking University, Beijing, China

**Keywords:** prediction model, machine learning, mechanical ventilation, intensive care unit, death

## Abstract

**Background:** Mechanically ventilated patients in the intensive care unit (ICU) have high mortality rates. There are multiple prediction scores, such as the Simplified Acute Physiology Score II (SAPS II), Oxford Acute Severity of Illness Score (OASIS), and Sequential Organ Failure Assessment (SOFA), widely used in the general ICU population. We aimed to establish prediction scores on mechanically ventilated patients with the combination of these disease severity scores and other features available on the first day of admission.

**Methods:** A retrospective administrative database study from the Medical Information Mart for Intensive Care (MIMIC-III) database was conducted. The exposures of interest consisted of the demographics, pre-ICU comorbidity, ICU diagnosis, disease severity scores, vital signs, and laboratory test results on the first day of ICU admission. Hospital mortality was used as the outcome. We used the machine learning methods of *k*-nearest neighbors (KNN), logistic regression, bagging, decision tree, random forest, Extreme Gradient Boosting (XGBoost), and neural network for model establishment. A sample of 70% of the cohort was used for the training set; the remaining 30% was applied for testing. Areas under the receiver operating characteristic curves (AUCs) and calibration plots would be constructed for the evaluation and comparison of the models' performance. The significance of the risk factors was identified through models and the top factors were reported.

**Results:** A total of 28,530 subjects were enrolled through the screening of the MIMIC-III database. After data preprocessing, 25,659 adult patients with 66 predictors were included in the model analyses. With the training set, the models of KNN, logistic regression, decision tree, random forest, neural network, bagging, and XGBoost were established and the testing set obtained AUCs of 0.806, 0.818, 0.743, 0.819, 0.780, 0.803, and 0.821, respectively. The calibration curves of all the models, except for the neural network, performed well. The XGBoost model performed best among the seven models. The top five predictors were age, respiratory dysfunction, SAPS II score, maximum hemoglobin, and minimum lactate.

**Conclusion:** The current study indicates that models with the risk of factors on the first day could be successfully established for predicting mortality in ventilated patients. The XGBoost model performs best among the seven machine learning models.

## Introduction

Mechanically ventilated patients account for more than a quarter in the intensive care unit (ICU) (1). Invasive mechanical ventilation is associated with multiple complications and high mortality (2). The mechanical ventilation ratio has been increasing in the ICU in recent years due to the aging population, more survivors with cancers and comorbidities, and the advancements in treatment (3, 4).

Prediction models are useful tools to unearth underlying causes and provide assistance for clinical practice (5). Establishing a death prediction model of mechanically ventilated patients using their early-stage, easily obtained, and well-generalized features might be helpful for ICU physicians for early alerting and judgment.

With the development of machine learning algorithms, modeling methods are more diversified (6, 7). Extreme Gradient Boosting (XGBoost) has been widely recognized and highly praised in a number of data mining challenges (8–10). With its notable advantages, we hypothesized that the XGBoost model would perform better than other models. We planned to develop and validate multiple machine learning models using the data available in the early stages to predict hospital mortality and identify risk factors in mechanically ventilated ICU patients.

## Methods

### Database and Study Design

The Medical Information Mart for Intensive Care (MIMIC-III) database was used as the data resource (11). MIMIC-III is a single-center database covering 38,597 distinct adult patients admitted to the ICU in the Beth Israel Deaconess Medical Center in Boston from 2001 to 2012. MIMIC-III integrates comprehensive clinical data and makes them accessible to researchers worldwide under data use agreement. We have obtained permission after application and completion of the course and test (record IDs: 32994435 and 32450965). We established and validated the prediction models using the retrospectively extracted data in MIMIC-III. This study was performed based on the transparent reporting of a multivariable prediction model for individual prognosis or diagnosis (TRIPOD) guideline (12).

### Subjects, Variables, and the Outcome Extraction

Adult ICU patients treated with invasive mechanical ventilation during ICU stay were included. Subjects aged younger than 18 years or older than 90 years or who lack information on the outcome measure were excluded. Hospital mortality was used as the outcome measure.

The subject IDs were used to identify distinct adult patients. The predictors included: (a) demographic information: age and gender; (b) medical history: uncomplicated hypertension (defined as hypertension without complication), complicated hypertension (defined as hypertension with complication), uncomplicated diabetes (defined as diabetes without complication), complicated diabetes (defined as diabetes with complication), malignancy, hematologic disease, metastasis, peripheral vascular disease, hypothyroidism, chronic heart failure, stroke, and liver disease; (c) disease severity score: Simplified Acute Physiology Score II (SAPS II), Sequential Organ Failure Assessment (SOFA), and Oxford Acute Severity of Illness Score (OASIS); (d) diagnosis: sepsis, any organ failure, severity of respiratory failure, severity of coagulation failure, severity of liver failure, severity of cardiovascular failure, severity of central nervous system failure, severity of renal failure, respiratory dysfunction, cardiovascular dysfunction, renal dysfunction, hematologic dysfunction, metabolic dysfunction, and neurologic dysfunction; (e) vital signs on the first day of ICU admission: the highest, lowest, and mean levels of heart rate (HR), mean arterial pressure (MAP), systolic blood pressure (SBP), diastolic blood pressure (DBP), and temperature; and (f) laboratory results of the first day of ICU admission: the highest, lowest, and mean levels of lactate, pH, glucose, white blood cell (WBC), blood urea nitrogen (BUN), creatinine, and hemoglobin. Treatment information on renal replacement therapy (RRT) and the duration of mechanical ventilation were extracted to present the characteristics of the included subjects; they were not analyzed as predictors since we included only early-stage predictors, which can be obtained on the first day of ICU admission in this prediction model. The lengths of stay in hospital of survivors and non-survivors were reported. The target subjects together with all the predefined predictors, subject ID, characteristic variables, and the outcome measure were extracted using a Structured Query Language (SQL) script. The definition of the medical condition was referred to the ICD-9 code (13) and derived from the GitHub (https://github.com/MIT-LCP/mimic-code). The severity of respiratory, coagulation, liver, cardiovascular, central nervous system, or renal failure referred to the SOFA score of the specific organ (scores 0–4). The first day indicates the first 24 h of ICU admission. The SOFA, SAPS II, and OASIS scores refer to the first scores after ICU admission. After the extraction of the data, subjects who met the exclusion criteria were excluded. Then, the extreme and error values failing the logic check were censored. We excluded variables with missing values accounting for more than 30% of the sample size (14). Otherwise, we used the mean imputation method to deal with missing values. Thus, the subset was established for the final analyses.

### Statistical Analysis

The characteristics of the included patients were compared between survivors and non-survivors. The continuous variables are presented as the median and interquartile range (IQR) and compared using the *t*-test. The counting data are presented as numbers and percentages and compared using the chi-square test.

We employed seven machine learning methods—*k*-nearest neighbors (KNN), logistic regression, bagging, decision tree, random forest, XGBoost, and neural network—for model establishment. A sample of 70% of the cohort generated randomly using a seed was applied for the training set; the remaining 30% was used for testing. Areas under the receiver operating characteristic curves (AUCs) were used to evaluate the performance of the models. Calibration plots were drawn to visualize the prediction abilities of the models. For the best-performing model, the significance of the model parameters was identified and reported; the Shapley additive explanation (SHAP) plot was drawn. SAS software (version 9.4), R software (version 3.6.1), and Python software (version 3.4.3) were used for statistical analyses.

## Results

### Participants

**Figure 1:**
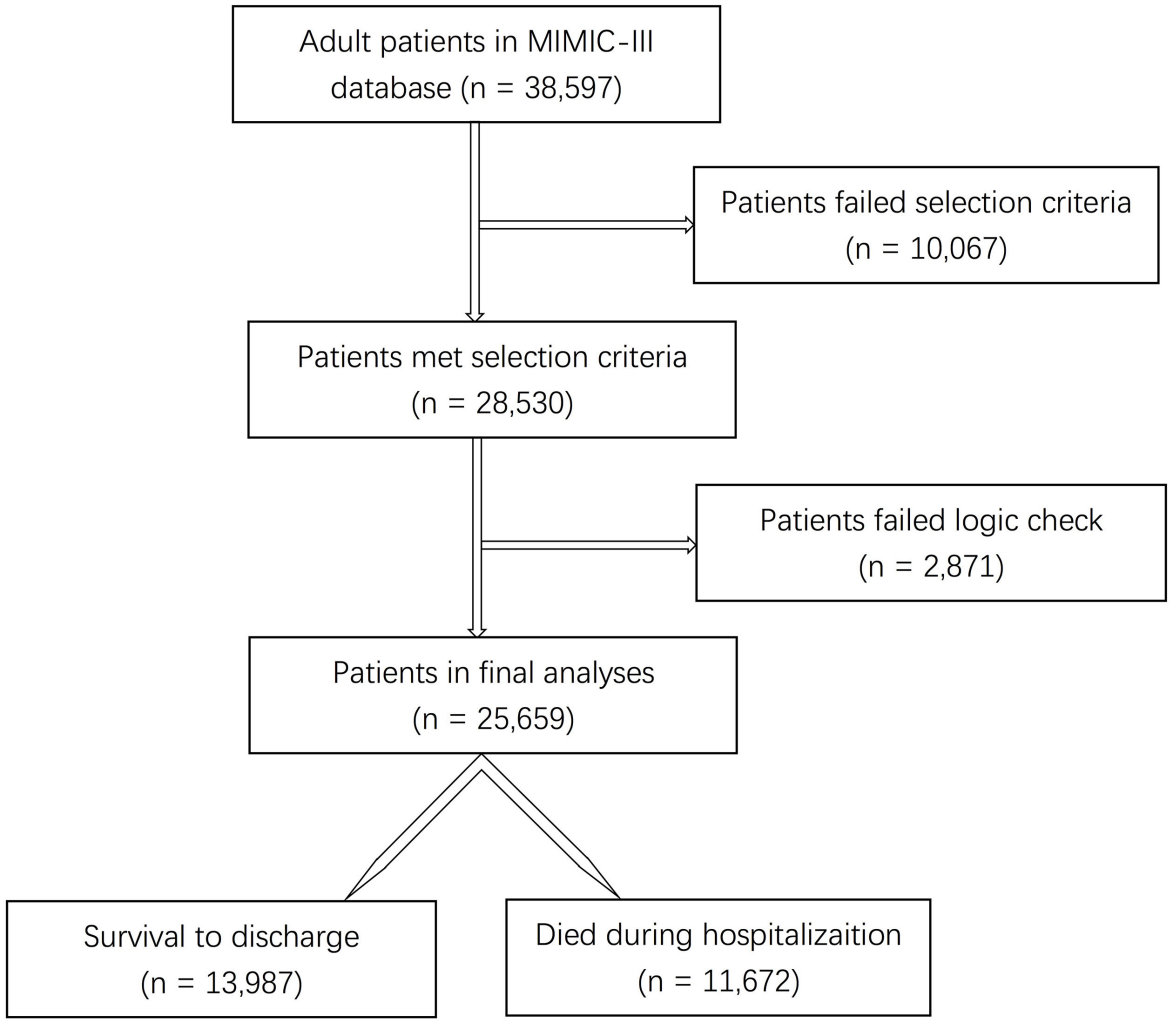
Flow diagram of the selection process of patients.

Among the 38,597 adult patients in the MIMIC-III database, 28,530 subjects met our selection criteria. After the logic check, 25,659 patients were included in the final analyses (Figure 1). Sixty-seven predictors were extracted from the database. After data cleaning, the predictor severe liver failure was excluded because of more than 30% of missing data; 66 predictors were included in the model. The mortality rate of the cohort was 45.5% (13,987 survivors and 11,672 non-survivors). The median length of stay in hospital of survivors was 9.2 days (IQR = 11.1) and that of non-survivors was 11.1 days (IQR = 15.3, *p* < 0.0001). The comparison of characteristics between the survivors and the non-survivors is reported in Table 1. Non-survivors were older and had higher SAPS II, SOFA, and OASIS scores; more medical history of hypertension with complication, diabetes with complication, malignancy, hematologic disease, peripheral vascular disease, hypothyroidism, chronic heart failure, stroke, and liver disease; more diagnosis of sepsis, any organ failure, severe respiratory failure, severe coagulation failure, severe liver failure, severe cardiovascular failure, severe central nervous system failure, severe renal failure, respiratory dysfunction, cardiovascular dysfunction, renal dysfunction, hematologic dysfunction, metabolic dysfunction, and neurologic dysfunction; had higher mean HR, maximum HR, maximum MAP, maximum SBP, mean lactate, minimum lactate, mean glucose, minimum glucose, maximum glucose, mean WBC, minimum WBC, maximum WBC, mean creatinine, minimum creatinine, and maximum creatinine; and had longer duration of mechanical ventilation and more RRTs (*p* < 0.05), while they had a lower male ratio, hypertension without complication, mean MAP, minimum MAP, mean SBP, minimum SBP, mean DBP, minimum DBP, mean temperature, maximum temperature, mean hemoglobin, minimum hemoglobin, and maximum hemoglobin (*p* < 0.05). There were no significant differences in diabetes without complication (*p* = 0.0815) and maximum DBP (*p* = 0.0636) between the two groups.

**Table 1:** Characteristics between survivors and non-survivors.

	**Survivors (*N* = 13,987)**	**Non-survivors (*N* = 11,672)**	***p*-value**
**Demographic**			
Age (years)	61.0 (21.9)	70.3 (20.4)	<0.0001
Gender (male)	8,681 (62.1)	6,728 (57.6)	<0.0001
**Medical history**			
Uncomplicated hypertension	6,888 (49.3)	4,346 (37.2)	<0.0001
Complicated hypertension	1,098 (7.9)	1,655 (14.2)	<0.0001
Uncomplicated diabetes	2,938 (21.0)	2,557 (21.9)	0.0815
Complicated diabetes	730 (5.2)	908 (7.8)	<0.0001
Malignancy	894 (6.4)	2,167 (18.6)	<0.0001
Hematologic disease	1,296 (9.3)	1,884 (16.1)	<0.0001
Metastasis	1,142 (8.2)	1,762 (15.1)	<0.0001
Peripheral vascular disease	1,225 (8.8)	1,142 (9.8)	0.0049
Hypothyroidism	1,217 (8.7)	1,172 (10.0)	0.0002
Chronic heart failure	744 (5.3)	780 (6.7)	<0.0001
Stroke	731 (5.2)	725 (6.2)	0.0007
Liver disease	616 (4.4)	919 (7.9)	<0.0001
**Disease severity**			
SAPS II	32.0 (16.0)	43.0 (19.0)	<0.0001
SOFA	4.0 (4.0)	5.0 (5.0)	<0.0001
OASIS	33.0 (10.0)	37.0 (12.0)	<0.0001
**Diagnosis**			
Sepsis	1,617 (11.6)	3,375 (28.9)	<0.0001
Any organ failure	8,150 (58.3)	9,920 (85.0)	<0.0001
Severe respiratory failure	659 (5.7)	966 (10.9)	<0.0001
Severe coagulation failure	27 (0.2)	149 (1.3)	<0.0001
Severe liver failure	101 (2.0)	323 (5.2)	<0.0001
Severe cardiovascular failure	1,070 (7.7)	2,116 (18.3)	<0.0001
Severe central nervous system failure	711 (5.1)	608 (5.3)	<0.0001
Severe renal failure	398 (2.9)	1,178 (10.1)	<0.0001
Respiratory dysfunction	6,172 (44.1)	8,478 (72.6)	<0.0001
Cardiovascular dysfunction	1,388 (9.9)	2,687 (23.0)	<0.0001
Renal dysfunction	2,934 (21.0)	5,103 (43.7)	<0.0001
Hematologic dysfunction	1,296 (9.3)	1,884 (16.1)	<0.0001
Metabolic dysfunction	1,142 (8.2)	1,764 (15.1)	<0.0001
Neurologic dysfunction	1,245 (8.9)	1,371 (11.8)	<0.0001
**Vital signs**			
Mean HR (bpm)	85.7 (17.9)	86.8 (22.1)	<0.0001
Minimum HR (bpm)	71.0 (18.0)	71.0 (21.0)	<0.0001
Maximum HR (bpm)	103.0 (25.0)	106.0 (29.0)	<0.0001
Mean MAP (mmHg)	76.7 (11.9)	75.1 (13.9)	<0.0001
Minimum MAP (mmHg)	59.0 (12.0)	55.7 (15.0)	<0.0001
Maximum MAP (mmHg)	101.7 (22.0)	102.0 (25.0)	<0.0001
Mean systolic pressure (mmHg)	115.0 (17.9)	113.9 (22.5)	<0.0001
Minimum systolic pressure (mmHg)	89.0 (18.0)	86.0 (22.0)	<0.0001
Maximum systolic pressure (mmHg)	148.0 (28.0)	149.0 (33.0)	<0.0001
Mean diastolic pressure (mmHg)	59.9 (11.6)	57.5 (13.2)	<0.0001
Minimum diastolic pressure (mmHg)	45.0 (12.0)	41.0 (15.0)	<0.0001
Maximum diastolic pressure (mmHg)	80.0 (19.0)	80.0 (22.0)	0.0636
Mean temperature (°C)	37.0 (0.8)	36.8 (0.9)	<0.0001
Minimum temperature (°C)	36.1 (1.0)	36.1 (1.0)	<0.0001
Maximum temperature (°C)	37.7 (1.0)	37.6 (1.1)	<0.0001
**Laboratory results**			
Mean lactate (mmol/L)	1.9 (1.2)	2.0 (1.9)	<0.0001
Minimum lactate (mmol/L)	1.3 (0.8)	1.5 (1.2)	<0.0001
Maximum lactate (mmol/L)	2.4 (2.0)	2.4 (2.8)	<0.0001
Mean pH	7.4 (0.1)	7.4 (0.1)	<0.0001
Minimum pH	7.3 (0.1)	7.3 (0.2)	<0.0001
Maximum pH	7.4 (0.1)	7.4 (0.1)	<0.0001
Mean glucose (mg/dL)	128.6 (32.1)	136.7 (50.2)	<0.0001
Minimum glucose (mg/dL)	96.0 (35.0)	104.0 (44.0)	<0.0001
Maximum glucose (mg/dL)	169.0 (60.0)	174.0 (86.0)	<0.0001
Mean WBC (×10^9^/L)	11.7 (5.9)	11.8 (7.6)	<0.0001
Minimum WBC (×10^9^/L)	9.8 (5.5)	10.1 (6.9)	<0.0001
Maximum WBC (×10^9^/L)	13.4 (7.3)	13.4 (8.9)	<0.0001
Mean BUN (mg/dl)	15.5 (10.3)	24.5 (24.0)	<0.0001
Minimum BUN (mg/dl)	14.0 (9.0)	23.0 (22.0)	<0.0001
Maximum BUN (mg/dl)	17.0 (11.0)	26.0 (25.0)	<0.0001
Mean creatinine (mg/dl)	0.9 (0.4)	1.1 (1.0)	<0.0001
Minimum creatinine (mg/dl)	0.8 (0.4)	1.0 (0.9)	<0.0001
Maximum creatinine (mg/dl)	0.9 (0.5)	1.2 (1.2)	<0.0001
Mean hemoglobin (g/dl)	10.6 (2.5)	10.3 (2.3)	<0.0001
Minimum hemoglobin (g/dl)	9.5 (3.0)	9.4 (2.6)	<0.0001
Maximum hemoglobin (g/dl)	12.4 (2.6)	11.3 (2.6)	<0.0001
**Treatment**			
Ventilation duration (h)	15.0 (45.9)	46.0 (122.6)	<0.0001
RRT	654 (4.7)	1,628 (14.0)	<0.0001

*Continuous variables are presented as the median and interquartile range (IQR). Counting data are presented as numbers and percentages*.

*Complicated or uncomplicated hypertension refers to hypertension with or without complication. Complicated or uncomplicated diabetes refers to diabetes with or without complication. Severe respiratory failure, severe coagulation failure, severe liver failure, severe cardiovascular failure, severe central nervous failure, and severe renal failure refer to the scores of the specific organ or system that reaches 4 in the SOFA score. The definition of the medical condition was referred to the ICD-9 code. A mean, minimum, or maximum parameter refers to the mean, the highest, or the lowest level of the parameter on the first day of ICU admission*.

*HR, heart rate; MAP, mean arterial pressure; OASIS, Oxford Acute Severity of Illness Score; RRT, renal replacement therapy; SAPS II, Simplified Acute Physiology Score II; SOFA, Sequential Organ Failure Assessment; WBC, white blood cell*.

### Models

With the training set, the KNN, logistic regression, decision tree, random forest, neural network, bagging, and XGBoost models were established and the testing set obtained AUCs of 0.806, 0.818, 0.743, 0.819, 0.780, 0.803, and 0.821, respectively.

**Figure 2:**
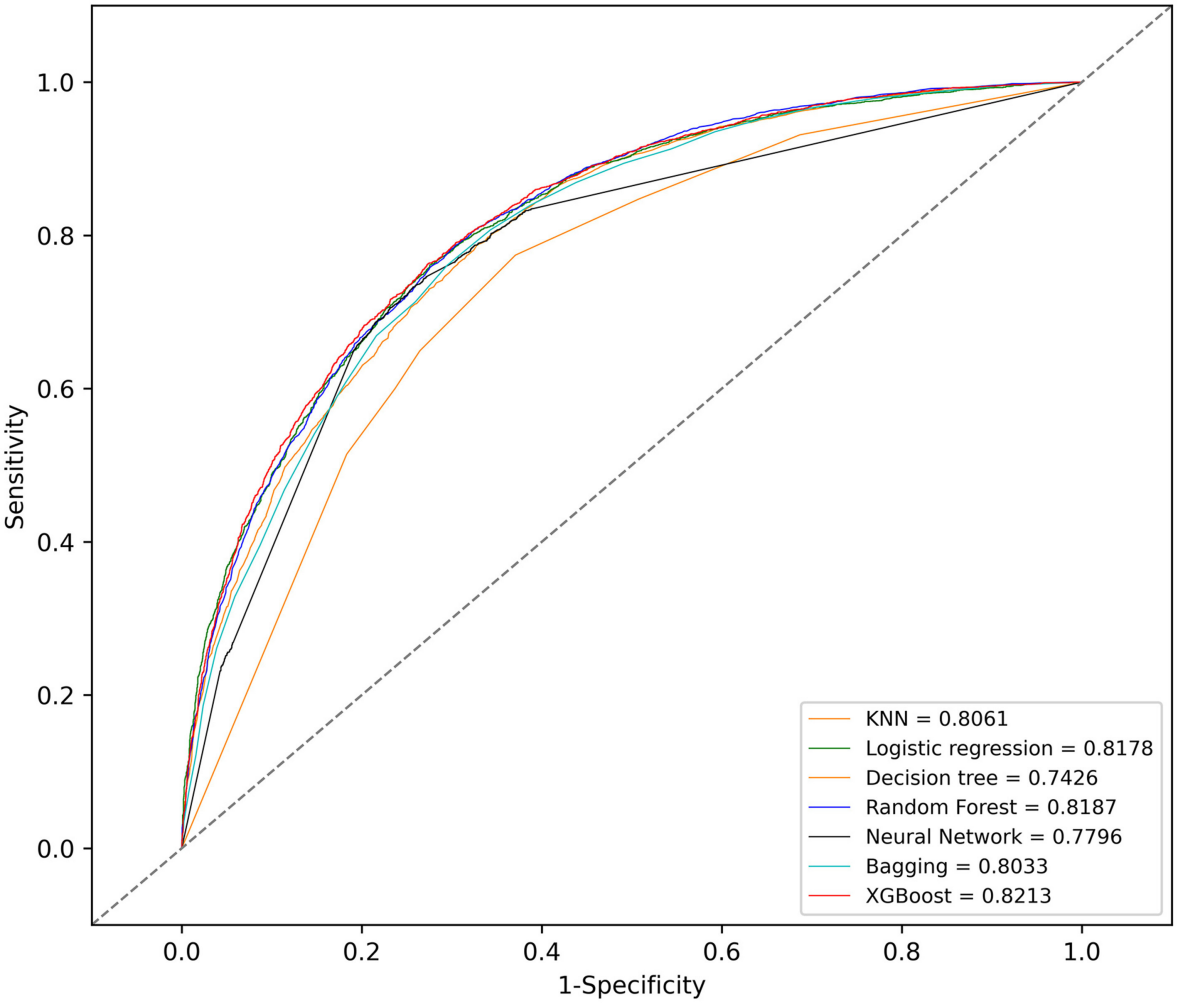
Receiver operating characteristic (ROC) curves of the seven models. KNN, k-nearest neighbors; XGBoost, Extreme Gradient Boosting.

**Figure 3:**
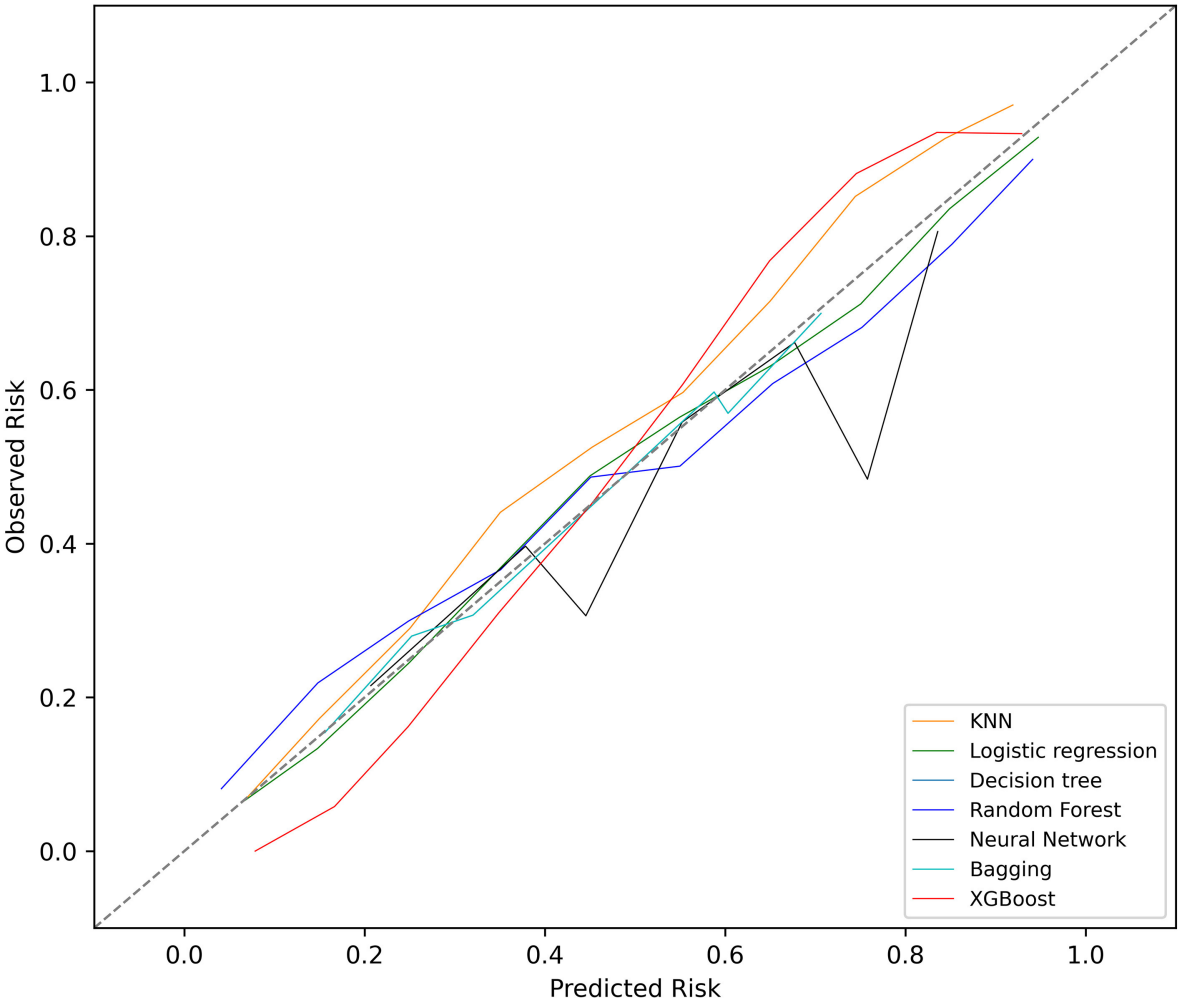
Calibration plots of the seven models. KNN, k-nearest neighbors; XGBoost, Extreme Gradient Boosting.

**Figure 4:**
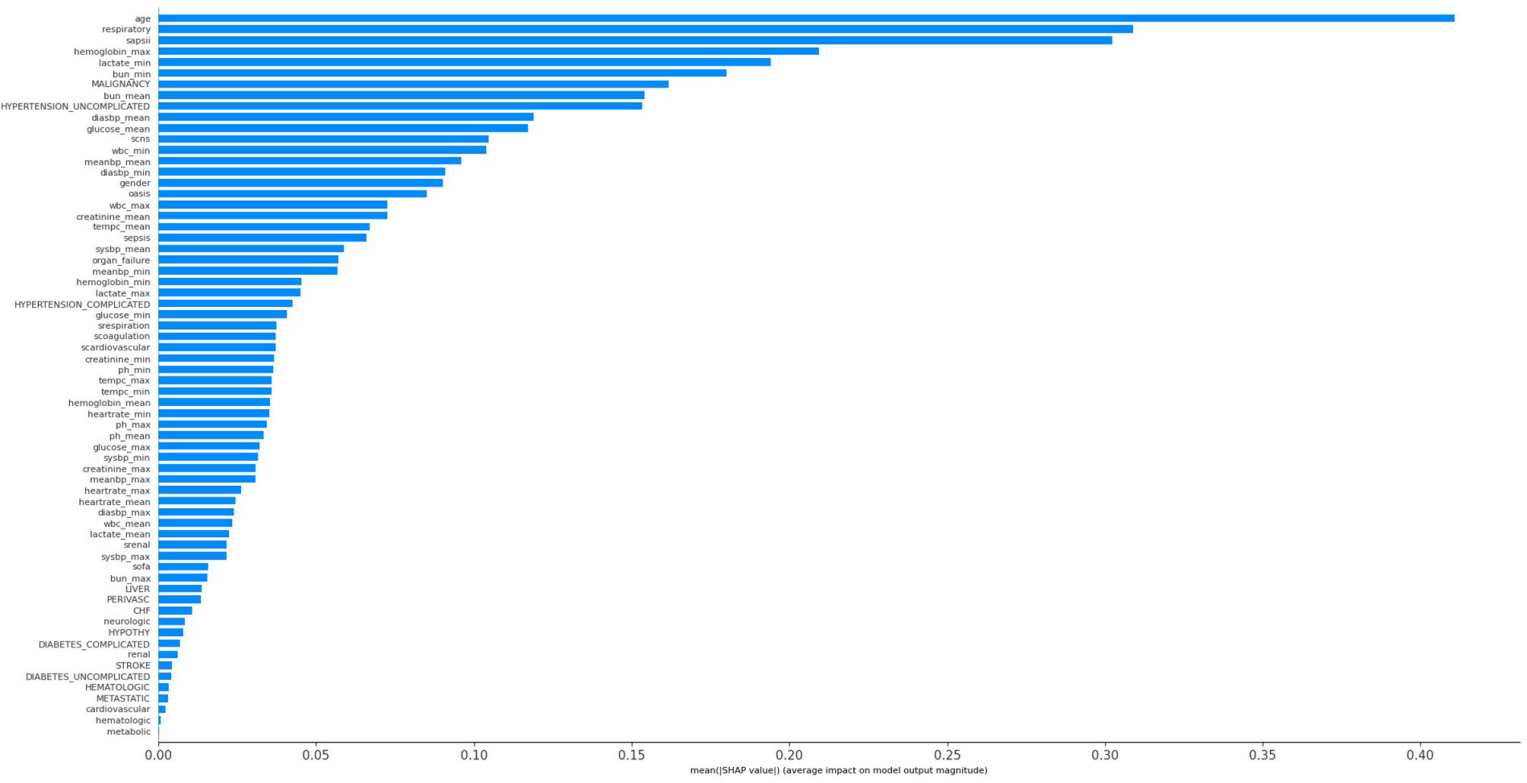
Significance of the predictors in the XGBoost model. CHF, chronic heart failure; Diabetes_complicated, diabetes with complication; Diabetes_uncomplicated, diabetes without complication; Diasbp, diastolic blood pressure; Hypertension_complicated, hypertension with complication; Hypertension_uncomplicated, hypertension without complication; OASIS, Oxford Acute Severity of Illness Score; Organ_failure, any organ failure; Perivasc, perivascular disease; SAPS II, Simplified Acute Physiology Score II; sCardiovascular, severe cardiovascular failure; sCNS, severe central nervous system failure; sCoagulation, severe coagulation failure; SOFA, Sequential Organ Failure Assessment; sRenal, severe renal failure; sRespiration, severe respiratory failure; Sysbp, systolic blood pressure; Tempc, temperature; WBC, white blood cell.

The KNN, logistic regression, decision tree, random forest, neural network, bagging, and XGBoost models were established with the training set; the AUCs of the testing set were 0.806, 0.818, 0.743, 0.819, 0.780, 0.803, and 0.821, respectively (Figure 2). The calibration plots of the seven models are presented in Figure 3. The calibration curves of all the models, except that of the neural network, performed well. Among the seven models, XGBoost performed best, with the highest receiver operating characteristic (ROC) and the best calibration curve. The hyperparameters applied in the final XGBoost model were as follows: learning rates = 0.008, number of estimators = 800, maximum depth of a tree = 6, α = 0, λ = 0. The significance of the predictors in the XGBoost model is presented in Figure 4. In the SHAP methodology, the top five predictors were age, respiratory dysfunction, SAPS II score, maximum hemoglobin, and minimum lactate (the importance values were 0.410, 0.309, 0.302, 0.209, and 0.194, respectively). The confusion matrix of the XGBoost model is presented in Table 2. The SHAP plot and a decision tree of the XGBoost model are in the Supplementary Material.

**Table 2:** Confusion matrix of the XGBoost model.

	**Precision**	**Recall**	**F1 score**
Survival	0.87	0.81	0.84
Death	0.66	0.74	0.70

## Discussion

This study identified various clinical features associated with increased hospital mortality among mechanically ventilated ICU patients. Through sophisticated machine learning methods, we determined that age, respiratory dysfunction, SAPS II score, maximum hemoglobin, and minimum lactate were most associated with hospital death. Among the seven models, XGBoost revealed the best performance in discrimination.

Our results showed that more than half of the ICU patients were under mechanical ventilation; the mortality of the mechanically ventilated patients was high (45.5%). The requirement for mechanical ventilation has increased in recent years (1). Therefore, it is of great importance to recognize early the patients at high risk of death with early-stage, well-generalized, and easily obtained features (15). With the development of machine learning algorithms, the magnitude of predictors that can be processed has mainly been largely enriched. Thus, advanced machine learning techniques allow researchers to establish more optimal models in comparison with conventional models (16). With such models, ICU physicians could be alerted early when patients become complicated and have deteriorated with mechanical ventilation.

A previous study conducted by Yao et al. (16) explored the death prediction model in postoperative septic patients using the MIMIC-III database. Similar to our results, they also found that the XGBoost model performed better in predicting hospital mortality than the other models. However, due to the different patient types and the various features included, the feature importance rankings were quite different (their top five predictors: fluid–electrolyte disturbance, coagulopathy, RRT, urine output, and cardiovascular surgery). Another study (5) used information from the first 24 h after admission to the ICU to build a 1-year death prediction model in septic patients based on the stochastic gradient boosting (SGB) methodology. The AUC of the SGB model was 0.8039, similar to the performance of XGBoost in our study. Both the SGB and XGBoost models belong to gradient boosting algorithms. Similar to our results, age ranked first in the feature importance (their top five predictors: age, urine output, maximum BUN, metastatic cancer, and maximum temperature).

There are strengths of our study. Firstly, this is the first study that established several advanced machine learning death prediction models focused on mechanically ventilated ICU patients. Secondly, we used MIMI-III, a high-quality database with a large sample size and comprehensive clinical information. Thirdly, we utilized advanced statistical methods, including seven machine learning models, with the 30% subset used for internal validation and the ROCs and calibration plots to evaluate the models (17).

There are limitations to our study. Firstly, our models were retrospectively established based on a single-center database. Thus, further prospective studies are needed to evaluate the generalization of our models and predictors. Secondly, there were missing data in our research. There was also a potential confounding variable that we were unable to assess because its missing data exceeded the predesigned limit. Thirdly, external validation has not been employed in this study; hence, the significance and evidence level were decreased. Fourthly, our study only focused on hospital mortality, while other important outcome measures such as ventilator-free days within 28 days and long-term mortalities still needed further investigation. Lastly, we did not exclude patients who were withdrawn from care, which may also provide bias.

## Conclusion

Our results suggest that age, respiratory dysfunction, SAPS II score, maximum hemoglobin, and minimum lactate might be closely associated with hospital mortality in mechanically ventilated ICU patients. The XGBoost model performs better than the KNN, logistic regression, bagging, decision tree, random forest, and neural network models in our study. Further external validations are needed to test the generalization of our models and predictors.

## Data Availability Statement

The datasets presented in this study can be found in online repositories. The names of the repository/repositories and accession number(s) can be found below: https://mimic.physionet.org.

## Ethics Statement

The establishment of this database was approved by the Massachusetts Institute of Technology (Cambridge, MA) and Beth Israel Deaconess Medical Center (Boston, MA), and consent was obtained for the original data collection. Therefore, the ethical approval statement and the need for informed consent were waived for this manuscript. Written informed consent for participation was not required for this study in accordance with the national legislation and the institutional requirements.

## Author Contributions

YZ and HH conceptualized the research aims, planned the analyses, and guided the literature review. YL and QY extracted the data from the MIMIC-III database. JZ, GW, GC, SL, XJ, and JG participated in processing the data and doing the statistical analysis. YZ wrote the first draft of the paper. RY, CR, HZ, YC, QG, LL, BD, XX, WL, and HH provided comments and approved the final manuscript. All authors read and approved the final manuscript.

## Conflict of Interest

XJ was employed by company Yidu Cloud Technology Inc. JG was employed by Beijing Big Eye Xing Tu Culture Media Co., Ltd. The remaining authors declare that the research was conducted in the absence of any commercial or financial relationships that could be construed as a potential conflict of interest.

## Abbreviations

AUCs: areas under the receiver operating characteristic curves
DBP: diastolic blood pressure
HR: heart rate
ICU: intensive care unit
KNN: *k*-nearest neighbors
MAP: mean arterial pressure
MIMIC-III: Medical Information Mart for Intensive Care
OASIS: Oxford Acute Severity of Illness Score
ROC: receiver operating characteristic
RRT: renal replacement therapy
SAPS II: Simplified Acute Physiology Score II
SHAP: Shapley additive explanation
SBP: systolic blood pressure
SGB: stochastic gradient boosting
SOFA: Sequential Organ Failure Assessment
SQL: Structured Query Language
WBC: white blood cell
XGBoost: Extreme Gradient Boosting.
