# Supplementary figures and images for: Machine Learning Prediction Models for Mechanically Ventilated Patients: Analyses of the MIMIC-III Database
Source: Front Med (Lausanne). 2021 Jul 1;8:662340. doi: 10.3389/fmed.2021.662340 (PMC8280779; doi:10.3389/fmed.2021.662340)

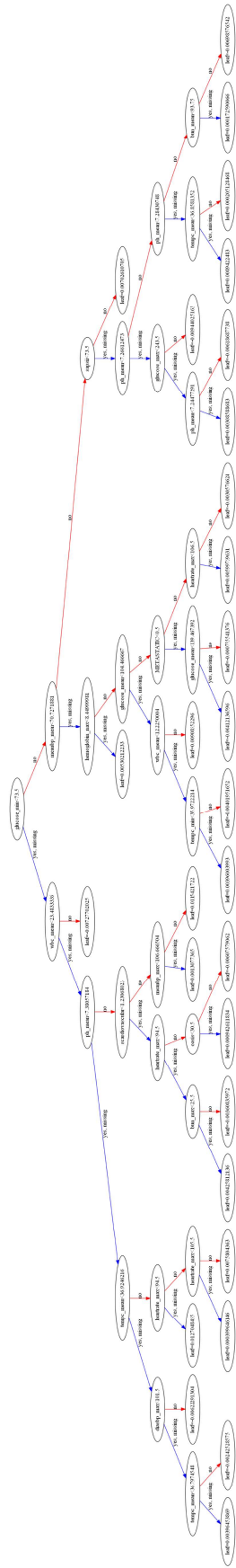

Supplement: Supplementary file 1 [file Data_Sheet_1.pdf]

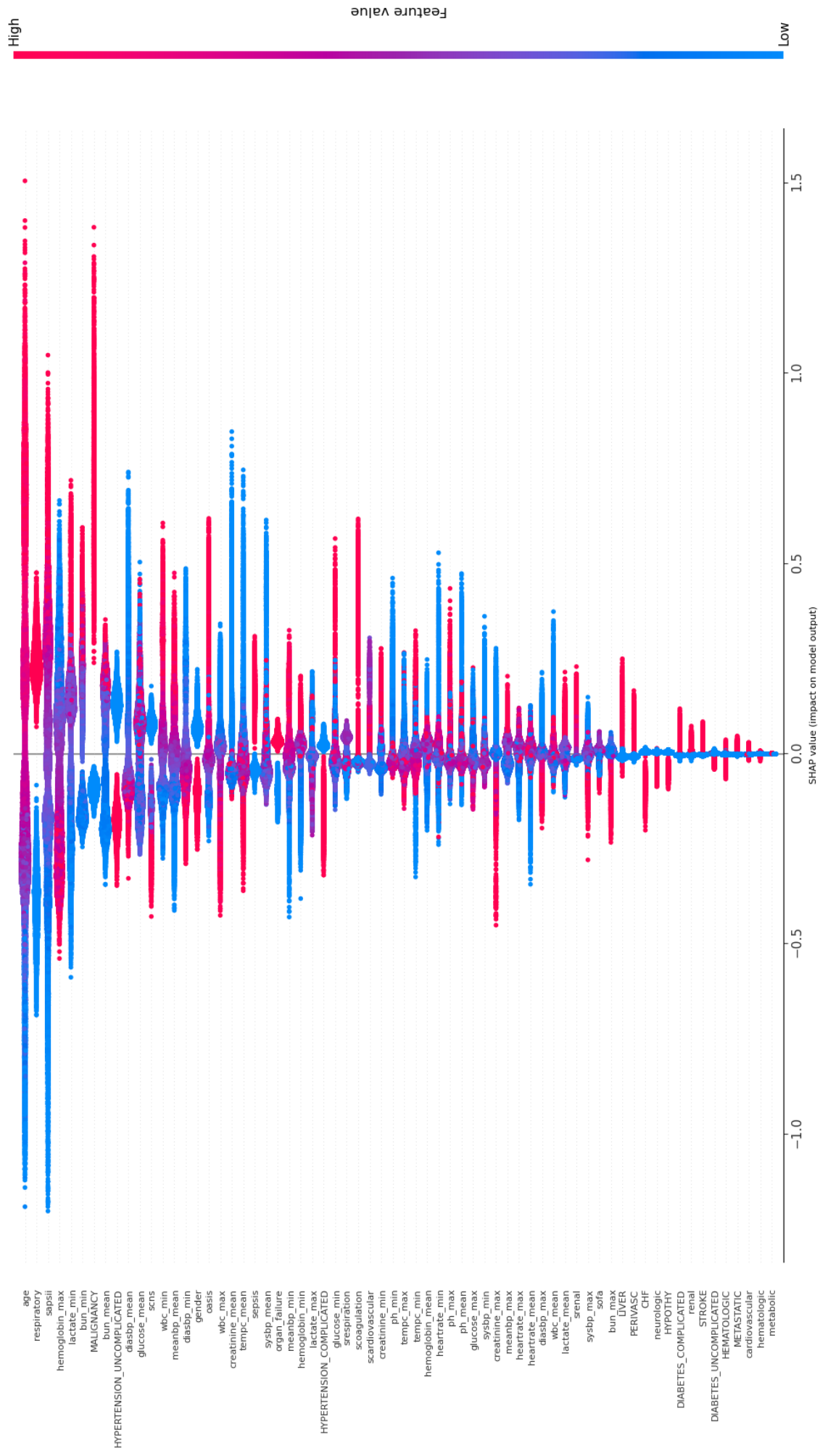

Supplement: Supplementary file 2 [file Data_Sheet_2.PDF]
